# Metagenomic analysis of the turkey gut RNA virus community

**DOI:** 10.1186/1743-422X-7-313

**Published:** 2010-11-12

**Authors:** J Michael Day, Linda L Ballard, Mary V Duke, Brian E Scheffler, Laszlo Zsak

**Affiliations:** 1 Southeast Poultry Research Laboratory Agricultural Research Service United States Department of Agriculture 934 College Station Road Athens, GA 30605 USA; 2 Genomics and Bioinformatics Research Unit Agricultural Research Service United States Department of Agriculture 141 Experiment Station Road Stoneville, MS 38776 USA

## Abstract

Viral enteric disease is an ongoing economic burden to poultry producers worldwide, and despite considerable research, no single virus has emerged as a likely causative agent and target for prevention and control efforts. Historically, electron microscopy has been used to identify suspect viruses, with many small, round viruses eluding classification based solely on morphology. National and regional surveys using molecular diagnostics have revealed that suspect viruses continuously circulate in United States poultry, with many viruses appearing concomitantly and in healthy birds. High-throughput nucleic acid pyrosequencing is a powerful diagnostic technology capable of determining the full genomic repertoire present in a complex environmental sample. We utilized the Roche/454 Life Sciences GS-FLX platform to compile an RNA virus metagenome from turkey flocks experiencing enteric disease. This approach yielded numerous sequences homologous to viruses in the BLAST nr protein database, many of which have not been described in turkeys. Our analysis of this turkey gut RNA metagenome focuses in particular on the turkey-origin members of the *Picornavirales*, the *Caliciviridae*, and the turkey Picobirnaviruses.

## Introduction

Enteric disease syndromes such as Poult Enteritis Complex (PEC) in young turkeys and Runting-Stunting Syndrome (RSS) in chickens are a continual economic burden for poultry producers. The only reliable method to reproduce the clinical signs of these syndromes in experimental birds is oral inoculation with crude preparations of intestinal contents from naturally infected birds. Further, the full spectrum of the field signs observed associated with these syndromes is difficult to reproduce experimentally with isolated viruses [1,2]. Numerous viruses are known to be circulating in turkey flocks in the United States, with recent research efforts targeting RNA viruses such as the turkey astroviruses, novel turkey-origin reovirus, and avian rotavirus, and DNA viruses such as the recently described turkey parvovirus [3-6]. However, there remains a possibility that an unidentified virus or combination of viruses may play a role in poultry enteric disease. Despite the isolation and characterization of many of these suspect viruses, the etiology of the poultry enteric disease syndromes remains elusive, and many enteric viruses can be detected in otherwise healthy turkey and chicken flocks [3,4]. Regional and national enteric virus surveys have revealed the ongoing presence of avian reoviruses, rotaviruses and astroviruses in turkey and chicken flocks, with combinations of viruses often present in the poultry gut [3,4]. A non-biased, comprehensive approach to virus discovery that would not require viral cultivation would reveal a great deal about the complex viral community in the turkey gut. Further, a community-based understanding of the viruses in the poultry gut will be an invaluable asset in ongoing studies of the enteric disease syndromes and would be welcome knowledge to poultry producers who rely upon efficient conversion of feed in the gut to produce an economically important commodity. A recent study utilizing a sequence-independent molecular screen of virus particle associated nucleic acid (PAN) in chicken enteric samples identified a novel chicken parvovirus (ChPV). This parvovirus is a member of the *Parvovirinae *sub-family within the *Parvoviridae*, and a PCR-based diagnostic test has been developed that targets the ChPV non-structural (NS) gene [6,7].The success of this PAN procedure suggests that similar approaches can be used for virus discovery in the poultry gut [7]. Ultra high-throughput nucleic acid pyrosequencing has emerged as a powerful diagnostic technology that can be applied to determine the full genomic repertoire present in a complex environmental sample [8]. Viral metagenomics can be specifically utilized to analyze viral sequences in just about any sample type, and is a powerful tool for virus discovery [9-13]. Further, viral metagenomics can be specifically applied to the problem of determining etiology in diseases and disease syndromes with no known cause [14-16]. In order to characterize the un-described viruses present in the turkey gut, we utilized the Roche/454 Life Sciences GS-FLX pyrosequencing platform to compile an RNA virus metagenome from turkeys experiencing enteric disease. The present analysis focused on RNA viruses in the turkey gut due to the large number of RNA viruses that have been identified to date as possibly contributing to enteric disease and poultry production problems. This approach yielded numerous sequences homologous to viruses in the National Center for Biotechnology Information (NCBI) BLAST non-redundant (nr) protein database, many of which have not been described in turkeys. These results validate this metagenomic approach to identifying known and novel RNA viruses in the poultry gut. The sequence data generated via this approach will prove useful in the molecular characterization of the viral constituency of the poultry gut, and will inform the selection of molecular diagnostic tests for enteric viruses. This will facilitate the development of updated molecular diagnostic tests, and a more thorough knowledge of the viral constituency in the poultry gut will lead to a better understanding of the role viruses play in enteric disease and in the performance of poultry flocks in general.

## Results and Discussion

**Figure 1:**
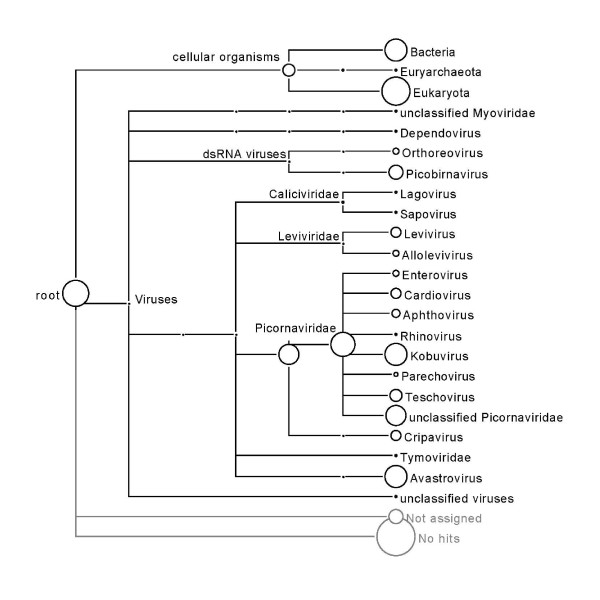
**MEGAN tree with taxonomic assignments**. The blastx output of the total contigs was assembled using the gsAssembler software. Circles located next to taxa are proportional to the total number of contigs identified in the pyrosequencing run and subsequent assembly (see Materials and Methods).

The initial pyrosequencing runs produced in excess of 139,000,000 bases of high quality nucleotide sequence with an average read length of 362. The sequence data was used to assemble 6526 contigs ranging in size from 97 to 2578 bp, with the majority of contigs falling in the range of approximately 250 to 450 bp. 4563 contigs produced no hits in the nr protein database using the blastx search parameters and the MEGAN default settings. 724 contigs had similarity to sequences from cellular organisms, including bacteria, fungi and avian species. 788 contigs had similarity to RNA viral sequences, including sequences from the dsRNA viruses (*Reoviridae *and Picobirnaviruses), and the ssRNA viruses (*Caliciviridae*, *Leviviridae*, *Picornavirales*, and *Astroviridae*) (Figure 1). The number of cellular sequences in the present dataset are likely due to the use of intestinal homogenates, which included intestinal tissue in order to ensure the discovery of cell-associated viruses in the submitted samples. The tblastx search output produced a MEGAN taxon tree similar to the one presented in Figure 1 and revealed that many of the unassigned contigs were similar to avian sequences.

The majority of the assigned viral contigs (620) showed similarity to database sequences from the *Picornavirales *order and other picorna-like viruses, viruses that, as a group, contain a positive sense single-stranded RNA genome and a virion approximately 30 nm in diameter [17]. Recently, a retrospective study of electron micrographs of enteric viruses from California turkeys experiencing enteric disease revealed a large number of "small round viruses" ranging in size from 15 to 30 nm, like most members of the *Picornavirales *[18]. These small round viruses are present in turkeys across a range of ages, but they have only been identified morphologically, making specific identification difficult. Avian enterovirus-like viruses have been described for years in domestic poultry; again this designation has historically been made based primarily upon morphological characterization, and little is known about their pathogenicity or their transmission characteristics[19]. It is unclear what role these picornaviruses and picorna-like viruses may play in turkey enteric disease or in turkey performance in general, but the presence of picornaviruses in other agricultural species has been closely associated with enteric disease, namely in pigs and cattle [20-23]. Members of the *Picornavirales *also infect avian species, with the etiologic agents of duck hepatitis (DH) in ducklings and avian encephalomyelitis (AE) in several poultry species both being picornaviruses [24,25]. The present metagenomic analysis has identified RNA sequences with homology to seven of the nine recognized picornavirus genera [26], with the largest proportion of the sequences bearing homology to the *Kobuvirus *genus.

**Figure 2:**
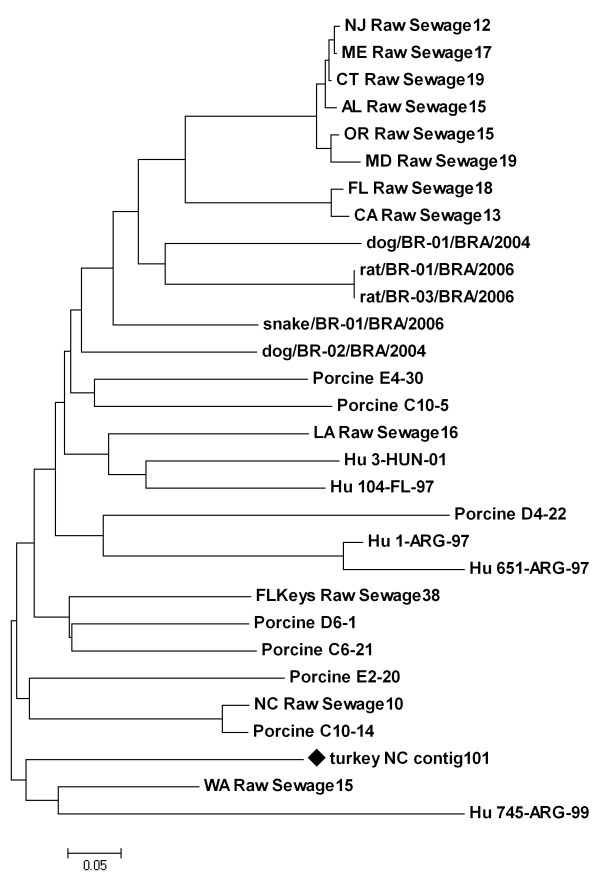
**Picobirnavirus neighbor-joining tree**. The evolutionary history for a 201 bp portion of the PBV RdRp gene was inferred using the Neighbor-Joining method, the optimal tree with the sum of branch length = 4.85654838 is shown. The nucleotide tree is drawn to scale, with branch lengths in the same units as those of the evolutionary distances used to infer the phylogenetic tree. The evolutionary distances were computed using the Kimura 2-parameter method and are in the units of the number of base substitutions per site. The turkey-specific sequence used in the analysis is indicated with a black diamond; its accession number is HM803965. Hu = Human.

The picobirnaviruses (PBVs) are a relatively recently described group of viruses that contain dsRNA, bi-segmented genomes and have non-enveloped capsids generally around 35 nm in diameter [27]. Since their initial description, the PBVs have been detected in enteric samples from several mammalian hosts, including humans [28-30]. A PBV has been described in chickens based upon morphological characterization and electropherotyping, along with a similar virus with an apparent tri-segmented genome [31]. The chicken PBV was not specifically associated with enteric disease. PBVs have been associated with gastroenteritis in humans [32,33], but a recent metagenomic analysis of the viral community in feces from healthy human volunteers revealed a relatively large number of PBV sequences [11]. Interestingly, PBVs have been detected in humans together with rotaviruses and astroviruses [32,34]. Phylogenetic analysis of a portion of the RNA-dependent RNA polymerase gene (RdRp) reveals that the putative turkey-origin PBV identified in the present metagenomic analysis is unique among the available PBV sequences, which include PBVs from humans, pigs, dogs, rats, snakes and municipal raw sewage (Figure 2). At the nucleotide level, the portion of the turkey-origin PBV used for the alignment and subsequent phylogenetic analysis shared from 49.5 to 70% sequence identity with the PBV sequences selected from the database; the highest identity was with the PBV detected in raw sewage in Washington state.

**Figure 3:**
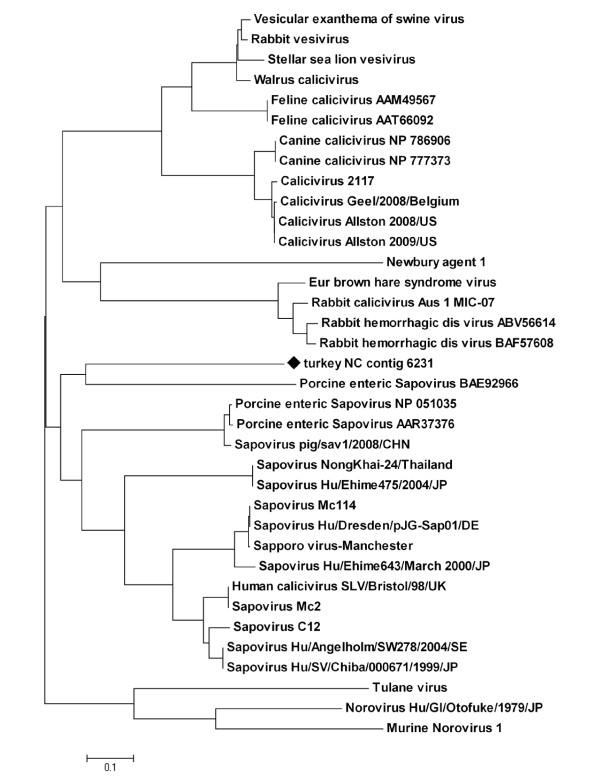
**Calicivirus neighbor-joining tree**. The evolutionary history of a ~300aa portion of the calicivirus ORF1 polyprotein was inferred using the Neighbor-Joining method. The optimal tree with the sum of branch length = 5.99935819 is shown. The tree is drawn to scale, with branch lengths in the same units as those of the evolutionary distances used to infer the phylogenetic tree. The evolutionary distances were computed using the Poisson correction method and are in the units of the number of amino acid substitutions per site. The turkey-specific sequence used in the analysis is indicated with a black diamond; its accession number is HM803966.

The family *Caliciviridae *includes four genera: *Norovirus*, *Sapovirus*, *Lagovirus*, and *Vesivirus *[26]. The present metagenomic analysis revealed nucleic acid with homology to database sequences from the *Sapovirus *and *Lagovirus *genera. The *Sapovirus *genus includes viruses that cause enteritis in swine and humans, and the Lagoviruses infect rabbits and hares (lagomorphs) [26]. In a phylogenetic analysis using a ~300 amino acid portion of ORF1 polyprotein homologous to the conserved P-loop NTPase superfamily, the putative turkey-origin Calicivirus grouped with the porcine enteric Sapoviruses (Figure 3). In general, the Sapoviruses are a very genetically heterogeneous genus [35], with numerous genogroups recognized, and many porcine Sapoviruses are related to human strains [36]; it will be interesting to determine the place the novel turkey caliciviruses hold in this genus, and it is notable that the portion of the ORF polyprotein analyzed in this study shared only 23.2 to 35% amino acid sequence identity with the Calicivirus isolates included in the alignment and subsequent phylogenetic analysis.

The appearance of avian reovirus and avian astrovirus sequences in the present analysis is not surprising, since these viruses are widespread in turkeys and chickens [3-5], and a good deal of recent research has helped to characterize the roles these viruses play in the poultry enteric syndromes [1,2,37-39]. The turkey astrovirus revealed using this metagenomic approach was most similar to the previously described type 2 turkey astroviruses (TAstV-2), which are very common in the turkey intestine [3]. The avian reovirus genes revealed using this approach--namely the lambdaA core protein gene, the non-structural protein muC, and the structural protein muB--have not been described in turkeys previously, but they have homology to previously described avian reovirus genome segments [40,41].

This first look at the turkey gut viral community using metagenomics has revealed a great deal of novel RNA virus sequence, and this analysis is a step toward identifying some of these undescribed, small enteric viruses. The dataset includes samples from areas that historically have had problems with enteric disease and includes a range of flock ages. The samples were collected regardless of the present enteric disease status of the flocks to ensure that the viral flora had not been perturbed by advanced enteric disease signs and due to the numerous observations that enteric viruses are often present in healthy birds [3,4]. The sequence data generated via this approach will prove useful in the molecular characterization of the viral constituency of the poultry gut, and will inform the selection of molecular diagnostic tests for enteric viruses. Further, this study sets the stage for subsequent comparative metagenomic analyses to determine the viruses commonly found in flocks with enteric syndromes versus the viral constituency of healthy flocks, for regional comparisons of circulating enteric viruses, and for comparing specific management and nutritional techniques and their effect on the gut microbiome.

## Materials and methods

### Gut samples receipt and preparation

With the cooperation of industry stakeholders, complete intestinal tracts (from duodenum/pancreas to cloaca, including cecal tonsils) from five turkey farms in North Carolina, U.S.A. with histories of enteric disease problems were received at the Southeast Poultry Research Laboratory in October 2008. Five complete intestinal tracts from each farm were collected. The intestines were processed promptly via blending into a ~20% homogenate in sterile phosphate-buffered saline (PBS) and were pooled into a single sample. This pooled intestinal homogenate represented turkeys ranging in age from 7 days to 34 days. After 5000 rpm (2400 × G) and 7500 rpm (5500 × G) centrifugation steps (15 min, 4°C) to clarify the homogenate (SLA 1500 SuperLite rotor, Sorvall), a stepwise filtration process involving 0.8 μm, 0.45 μm, and 0.2 μm cutoff bottle filters (Nalgene) was used to remove large particles and bacteria. Virus-sized particles were pelleted by ultracentrifugation (5 hr., 113,000 × G, 4°C).

### Viral RNA isolation and cDNA synthesis

The virus particle pellet was resuspended in Tris-HCl buffer (pH 7.5) and treated with RNAse A (Invitrogen) to remove unencapsidated (non-viral) RNA. Total RNA was extracted from the pellet using TRIZOL-LS reagent (Invitrogen) and RNA was further purified from the TRIZOL-LS aqueous phase using the MagMax Viral RNA isolation kit (Ambion)[42]. This RNA sample was treated with Turbo DNAse (Ambion) to minimize any remaining DNA. cDNA was generated with random hexamers using the Invitrogen SuperScript Choice System and was ligated to the included EcoRI/NotI double-stranded oligonucleotide adapters and sephacryl column purified per the manufacturer's instructions. PCR using an EcoRI/NotI adapter primer (5'-CGG CCG CGT CGA C-3') was used to amplify the cDNA. An initial PCR reaction of 50 μl was split into 5 equal reactions prior to thermal cycling (94°C for 30s, 55°C for 30s, 72°C for 2 min, times 35 cycles, followed by an extension at 72°C for 7 min). The reactions were pooled and purified (Qiagen MinElute PCR cleanup kit).

### High throughput sequencing and analysis

**Figure 4:**
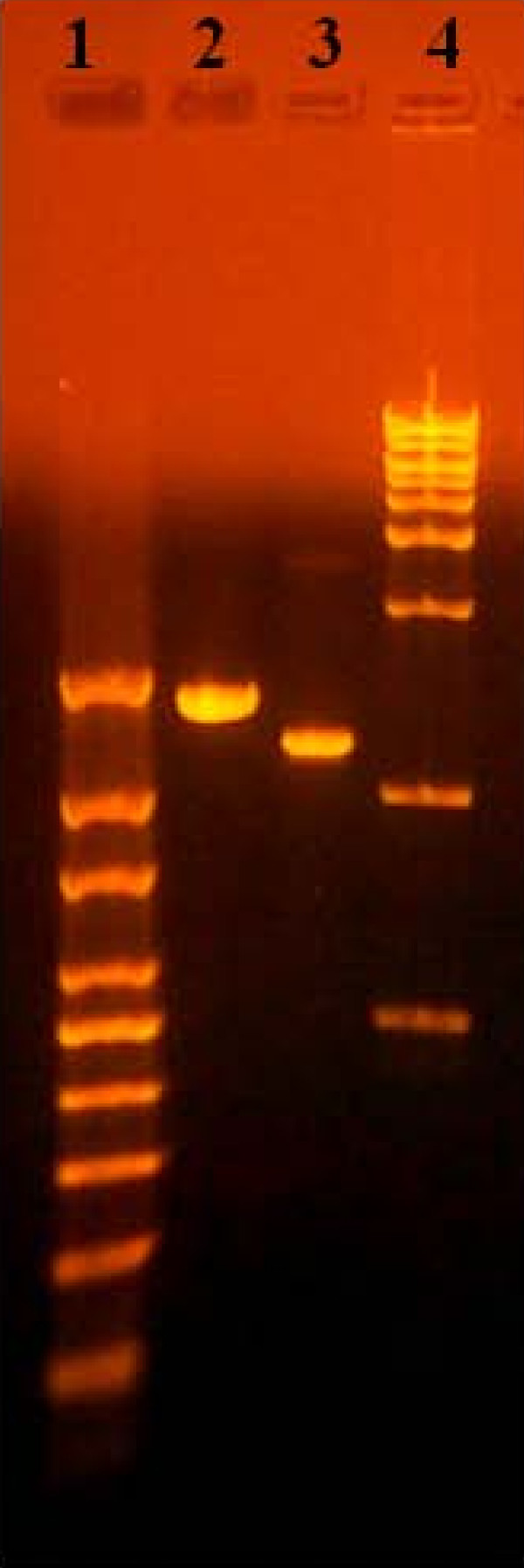
**Turkey picobirnavirus and calicivirus diagnostic agarose gel**. 1% agarose gel with RT-PCR amplicons from the turkey calicivirus ORF1 polyprotein region (1369 bp, lane 2) and the turkey picobirnavirus RdRp gene (1135 bp, lane 3). RNA was isolated from the intestinal contents collected from commercial North Carolina turkeys in 2009 (calicivirus positive) and 2010 (picornavirus positive).

The amplified cDNA was utilized in high-throughput nucleic acid sequencing using Genome Sequencer FLX Titanium pyrosequencing technology and reagents (Roche). Contigs were assembled using the gsAssembler software (454 Life Sciences) using stringent parameters (50 bp overlap with 95% identity). Using the assembled contigs as query sequences, the blast non-redundant (nr) protein database (GenBank) was searched using the blastx and tblastx programs. The blastx and tblastx output was compiled and contigs were assigned to taxa with MEGAN using the default LCA algorithm parameters [43]. Nucleotide and amino acid sequences were aligned using ClustalW and phylogenetic trees were prepared using MEGA4; the relationship of the sequences was inferred using the Neighbor-Joining method, which was determined to be a computationally efficient method to deal with datasets of this size and produced single trees to illustrate an initial placement of these novel viruses among available sequences [44]. In order to confirm directly the presence of picobirnavirus and calicivirus sequences in gut samples, the metagenomic contigs were utilized to design RT-PCR primers to amplify portions of the picobirnavirus RNA-dependent RNA polymerase (RdRp) and calicivirus ORF1 polyprotein. These primers were subsequently used to amplify viral sequences in the original RNA prep used to create the metagenome and in archived turkey intestinal samples from North Carolina, U.S.A (Figure 4).

## Competing interests

The authors declare that they have no competing interests.

## Authors' contributions

JMD conceived and coordinated the study, performed the virus isolation and cDNA production, and wrote the paper. LLB performed the bioinformatic analyses. MVD prepared the samples and performed the 454 pyrosequencing. BES coordinated the bioinformatic analyses and pyrosequencing. LZ provided technical input and intellectually contributed to the study design. All authors read and approved the final manuscript.
